# Fibrillization of Human Tau Is Accelerated by Exposure to Lead via Interaction with His-330 and His-362

**DOI:** 10.1371/journal.pone.0025020

**Published:** 2011-09-26

**Authors:** Hai-Li Zhu, Sheng-Rong Meng, Jun-Bao Fan, Jie Chen, Yi Liang

**Affiliations:** State Key Laboratory of Virology, College of Life Sciences, Wuhan University, Wuhan, China; University of Akron, United States of America

## Abstract

**Background:**

Neurofibrillary tangles, mainly consisted of bundles of filaments formed by the microtubule-associated protein Tau, are a hallmark of Alzheimer disease. Lead is a potent neurotoxin for human being especially for the developing children, and Pb^2+^ at high concentrations is found in the brains of patients with Alzheimer disease. However, it has not been reported so far whether Pb^2+^ plays a role in the pathology of Alzheimer disease through interaction with human Tau protein and thereby mediates Tau filament formation. In this study, we have investigated the effect of Pb^2+^ on fibril formation of recombinant human Tau fragment Tau_244–372_ and its mutants at physiological pH.

**Methodology/Principal Findings:**

As revealed by thioflavin T and 8-anilino-1-naphthalene sulfonic acid fluorescence, the addition of 5–40 µM Pb^2+^ significantly accelerates the exposure of hydrophobic region and filament formation of wild-type Tau_244–372_ on the investigated time scale. As evidenced by circular dichroism and Fourier transform infrared spectroscopy, fibrils formed by wild-type Tau_244–372_ in the presence of 5–40 µM Pb^2+^ contain more β-sheet structure than the same amount of fibrils formed by the protein in the absence of Pb^2+^. However, unlike wild-type Tau_244–372_, the presence of 5–40 µM Pb^2+^ has no obvious effects on fibrillization kinetics of single mutants H330A and H362A and double mutant H330A/H362A, and fibrils formed by such mutants in the absence and in the presence of Pb^2+^ contain similar amounts of β-sheet structure. The results from isothermal titration calorimetry show that one Pb^2+^ binds to one Tau monomer *via* interaction with His-330 and His-362, with sub-micromolar affinity.

**Conclusions/Significance:**

We demonstrate for the first time that the fibrillization of human Tau protein is accelerated by exposure to lead *via* interaction with His-330 and His-362. Our results suggest the possible involvement of Pb^2+^ in the pathogenesis of Alzheimer disease and provide critical insights into the mechanism of lead toxicity.

## Introduction

Alzheimer disease, a progressive and irreversible neurodegenerative disease, is the leading dementia in the elderly population (Approximately 10% of people over the age of 65) [1]. It has been reported that more than 90% of Alzheimer disease cases are sporadic despite several genetic mutations have been found in Alzheimer disease patients [2], [3]. Therefore, environmental exposure may be an etiologic factor in the pathogenesis of Alzheimer disease, either as triggers or as modulators of disease progression [2]. Among them, lead (Pb), a potent neurotoxin for human being, can be introduced into the organisms and may potentially modulate Alzheimer disease pathology because of the atmosphere emissions or the unhealthy workplaces especially in developing countries [4]. Exposure to lead mainly has a variety of adverse effects on the health of humans [4] especially for the developing children [5] whose central nervous system is sensitive and vulnerable to lead toxicity. Even exposure to low levels of inorganic lead (Pb^2+^) is known to induce lasting neurobehavioral and cognitive impairments [4], [5]. In addition, exposure to lead has been reported to associate with amyotrophic lateral sclerosis [6].

Alzheimer disease is characterized by the presence of senile plaques composed of amyloid β and neurofibrillary tangles. Neurofibrillary tangles are mainly consisted of bundles of filaments formed by the microtubule-associated protein Tau [7]. It has been reported that exposure to lead can increase amyloid precursor protein and amyloid β production in the aging brains of rodent [8] and primate [9], [10]. Meanwhile Pb^2+^ at high concentrations has been found in the brains of patients with Alzheimer disease [2] and with diffuse neurofibrillary tangles with calcification [11]. However, it has not been reported so far whether Pb^2+^ plays a role in the pathology of Alzheimer disease through interaction with human Tau protein and thereby mediates Tau filament formation.

Tau binds to microtubules through repeat domain in their C-terminal part [12]. Because the repeat domain of Tau forms the core of paired helical filaments in Alzheimer disease and also assembles more readily than full-length Tau into *bona fide* paired helical filaments *in vitro* [13], [14], we employed recombinant human Tau fragment Tau_244–372_ consisting of the four-repeat microtubule binding domain for studying kinetics of Tau fibril formation. In this study, we investigated the effect of Pb^2+^ on fibril formation of recombinant Tau_244–372_ and its mutants at physiological pH by using several biophysical methods, such as thioflavin T (ThT) binding, far-UV circular dichroism (CD), Fourier transform infrared (FTIR) spectroscopy, transmission electron microscopy (TEM), and isothermal titration calorimetry (ITC). We demonstrated for the first time that the fibrillization of human Tau protein was accelerated by exposure to 5–40 µM Pb^2+^ *via* interaction with His-330 and His-362, with sub-micromolar affinity. Our results suggest the possible involvement of Pb^2+^ in the pathogenesis of Alzheimer disease and provide important insights into the mechanism of lead toxicity.

## Materials and Methods

### Materials

Heparin (average MW = 6 kDa), ThT, and 8-anilino-1-naphthalene -sulfonic acid (ANS) were obtained from Sigma-Aldrich (St. Louis, MO). Dithiothreitol (DTT) was obtained from Ameresco (Solon, OH). All other chemicals used including Pb(NO_3_)_2_ were made in China and were of analytical grade.

### Plasmids and proteins

The cDNA encoding human Tau fragment Tau_244–372_ was amplified using the plasmid for human Tau40 (kindly provided by Dr. Michel Goedert) as a template. The PCR-amplified Tau_244–372_ was subcloned into pRK172 vector. Single histidine mutants H330A and H362A and double mutant H330A/H362A of Tau_244–372_ were generated using primers CACCTCCTGGTTTGGCATGGATGTT/AACATCCATGCCAAACCAGGAGGTG for H330A and GACAATATGCCCCACGTCCC/GGGACGTGGGGCATATTGTC for H362A. Triple mutant H268A/H299A/H329A and histidine-less mutant H268A/H299A/H329A/H330A/H362A were generated in a similar manner. Recombinant Tau_244–372_ and its mutants were expressed in *Escherichia coli* and purified to homogeneity by SP sepharose chromatography as described [15], [16]. Purified Tau protein was analyzed by SDS-PAGE with one band and confirmed by mass spectrometry. The concentration of human Tau fragment was determined according to its absorbance at 214 nm with a standard curve drawn by bovine serum albumin.

### Thioflavin T binding assays

A 2.5 mM ThT stock solution was freshly prepared in 10 mM HEPES buffer (pH 7.4) and passed through a 0.22-µm pore size filter before use to remove insoluble particles. Under standard conditions, 10 µM human Tau fragment was incubated in 10 mM HEPES buffer (pH 7.4) containing 100 mM NaCl, 1 mM DTT, and 20 µM ThT with or without Pb^2+^ at 37°C for up to 8 h in the presence of fibrillization inducer heparin used in a Tau ∶ heparin molar ratio of 4 ∶ 1. The solutions with a volume of 200 µl were placed into a well of a 96-well plate in SpectraMax M2 microplate reader (Molecular Devices, Sunnyvale, CA) using excitation at 440 nm and emission at 480 nm with a wavelength cut-off at 475 nm [16]. Each sample was run in triplicate or quadruplicate.

### Kinetic model

Kinetic parameters were determined by fitting ThT fluorescence intensity *versus* time to the empirical Hill equation [17]:
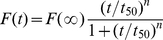
(1)where *F*(∞) is the fluorescence intensity in the long time limit, *t*_50_ is the elapsed time at which *F* is equal to one-half of *F*(∞), and *n* is a cooperativity parameter.

### ANS binding assays

A 2.5 mM ANS stock solution was freshly prepared in 10 mM HEPES buffer (pH 7.4) and passed through a 0.22-µm pore size filter before use to remove insoluble particles. Under standard conditions, 10 µM human Tau fragment was incubated in 10 mM HEPES buffer (pH 7.4) containing 1 mM DTT and 20 µM ANS with or without Pb^2+^ at 37°C for up to 1 h in the presence of heparin used in a Tau ∶ heparin molar ratio of 4 ∶ 1. The fluorescence of ANS was excited at 350 nm with a slit-width of 5.0 nm and the emission was 470 nm with a slit-width of 7.5 nm on an LS-55 luminescence spectrometer (PerkinElmer Life Sciences, Shelton, CT). Assays in the absence of the protein were performed to correct for unbound ANS emission fluorescence intensities.

### CD measurements

Under standard conditions, 10 µM human Tau fragment was incubated in 30 mM NaH_2_PO_4_-Na_2_HPO_4_ buffer (pH 7.4) (or 50 mM sodium acetate buffer at pH 7.4) containing 1 mM DTT and 2.5 µM heparin with or without Pb^2+^ at 37°C for up to 1 h. Circular dichroism spectra were obtained by using a Jasco J-810 spectropolarimeter (Jasco Corp., Tokyo, Japan) with a thermostated cell holder. Quartz cell with a 1 mm light-path was used for measurements in the far-UV region. Spectra were recorded from 195 to 250 nm for far-UV CD. The scan number for one sample was 45, and the scan time for each scan was about 74 s. No time interval was set between the sequential two scans. The final concentration of Tau_244–372_ was kept at 10 µM. The spectra of all scans were corrected relative to the buffer blank. The mean residue molar ellipticity [θ] (deg⋅ cm^2^⋅dmol^−1^) was calculated using the formula [θ] = (*θ*_obs_/10)(MRW/*lc*), where *θ*_obs_ is the observed ellipticity in deg, MRW the mean residue molecular weight (106.1 Daltons for Tau fragment), *l* the path length in cm, and *c* the protein concentration in g/ml.

### Fourier transform infrared spectroscopy

FTIR spectra of human Tau fibril samples were recorded in KBr pellets using a Nicolet 5700 FTIR spectrophotometer (Thermo Electron, Madison, WI). 200 µM human Tau fragment was incubated in 10 mM HEPES buffer (pH 7.4) containing 100 mM NaCl, 1 mM DTT, and 5 µM heparin with or without Pb^2+^ at 37°C for overnight. Then the samples were lyophilized (freeze drying) at −40°C for FTIR measurements. FTIR spectra were recorded in the range from 400 to 4000 cm^−1^ at 4 cm^−1^ resolution. The sample was scanned 128 times in each FTIR measurement, and the spectrum acquired is the average of all these scans. After FTIR assays, the spectra are analyzed by OMNIC 8 software to obtain FTIR second derivative spectra.

### Transmission electron microscopy

The formation of filaments by human Tau fragment was confirmed by electron microscopy of negatively stained samples. Sample aliquots of 10 µl were placed on copper grids, and left at room temperature for 1–2 min, rinsed with H_2_O twice, and then stained with 2% (w/v) uranyl acetate for another 1–2 min. The stained samples were examined using an H-8100 transmission electron microscope (Hitachi, Tokyo, Japan) operating at 100 kV.

### Isothermal titration calorimetry

ITC experiments on the interaction of Pb^2+^ with Tau_244–372_ and its mutants were carried out at 25.0°C using a VP-ITC titration calorimetry (MicroCal, Northampton, MA). Freshly purified Tau proteins (wild-type Tau_244–372_, single mutants H330A and H362A, double mutant H330A/H362A, triple mutant H268A/H299A/H329A, and histidine-less mutant H268A/H299A/H329A/H330A/H362A) were dialyzed against 50 mM Bis-Tris buffer (pH 7.4) containing 1 mM ethylenediaminetetraacetic acid (EDTA) and 100 mM NaCl, overnight at 4°C and then dialyzed against 50 mM Bis-Tris buffer (pH 7.4) containing 100 mM NaCl extensively to remove EDTA. A solution of 100 µM Tau protein was loaded into the sample cell (1.43 ml), and a solution of 1.5 mM Pb^2+^ was placed in the injection syringe (280 µl). The first injection (2 µl) was followed by 19–24 injections of 10 µl. Dilution heats of Pb^2+^ were measured by injecting Pb^2+^ solution into buffer alone and were subtracted from the experimental curves prior to data analysis. The stirring rate was 300 rpm. The resulting data were fitted to a single set of identical sites model using MicroCal ORIGIN software supplied with the instrument, and the standard molar enthalpy change for the binding, 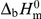, the dissociation constant, *K*_d_, and the binding stoichiometry, *n*, were thus obtained. The standard molar free energy change, 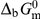, and the standard molar entropy change, 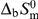, for the binding reaction were calculated by the fundamental equations of thermodynamics [16], [18]:
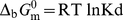
(2)

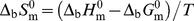
(3)


## Results

### The presence of Pb^2+^ enhanced Tau aggregation

The enhanced fluorescence emission of the dye ThT, a specific marker for the β-sheet conformation of fibril structures, has been widely used for monitoring the kinetics of amyloid fibril formation [16], [17], [19]. In order to mimic Tau fibrillization *in vivo*, heparin has been often employed to induce Tau filament formation *in vitro* [16]–[18], [20]. The kinetics for heparin-mediated Tau filament formation can be characterized by a lag period, followed by a period of exponential growth and an asymptotic approach to equilibrium [21].

**Figure 1:**
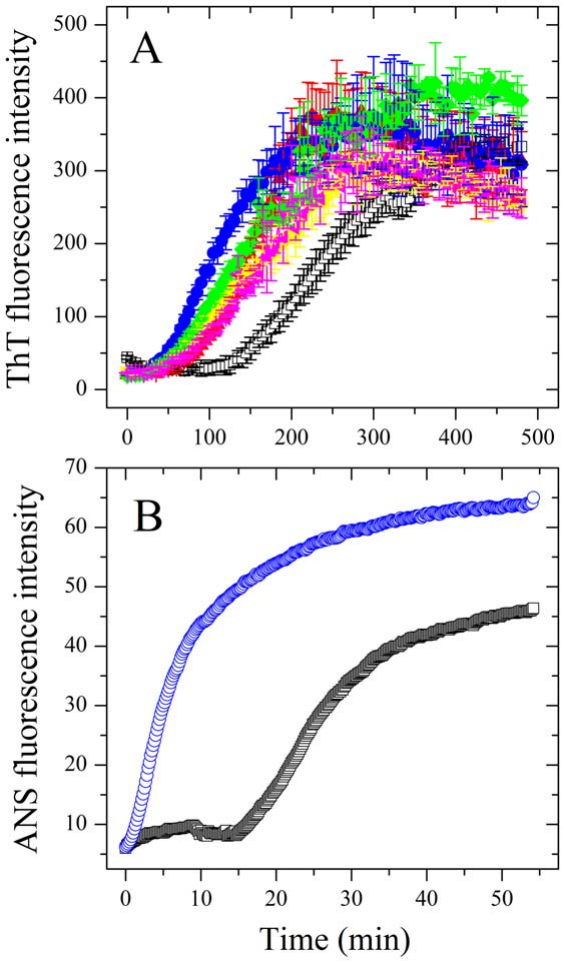
Pb^2+^ enhances Tau_244–372_ fibrillization. (A) 10 µM Tau_244–372_ was incubated with 0–40 µM Pb^2+^ (black: 0 µM, red: 5 µM, blue: 10 µM, yellow: 20 µM, green: 30 µM, and magenta: 40 µM) in 10 mM HEPES buffer (pH 7.4) containing 100 mM NaCl, 1 mM DTT, 2.5 µM heparin, and 20 µM ThT, and ThT binding assays were carried out at 37°C. Data are expressed as mean ± S.D. (*n* = 3–4). (B) 10 µM Tau_244–372_ was incubated with 0–10 µM Pb^2+^ (black: 0 µM, and blue: 10 µM) in 10 mM HEPES buffer (pH 7.4) containing 1 mM DTT, 2.5 µM heparin and 20 µM ANS, and ANS binding assays were carried out at 37°C.

In this paper, recombinant human Tau fragment Tau_244–372_ was incubated with Pb^2+^ ranging from 5 to 40 µM. Fitting human Tau fragment aggregation kinetic data (Fig. 1A) with the empirical Hill equation gave *t*_50_ and *F*(∞) values which reflect the lag phase and the final quantity of Tau_244–372_ amyloid formation respectively. The corresponding kinetic parameters are summarized in Table 1. As shown in Table 1, the value of *t*_50_ of Tau_244–372_ aggregation monitored by ThT binding assays were 143, 103, 142, 164, and 146 min in the presence of 5, 10, 20, 30, and 40 µM Pb^2+^ respectively, remarkably shorter than that in the absence of Pb^2+^ (255 min). The value of *t*_50_ reached the minimum at the molar ratio of Pb^2+^ to Tau of 1∶1, and then got longer at larger ratios of Pb^2+^/Tau (from 2∶1 to 4∶1). Therefore, as revealed by ThT binding assays (Fig. 1A and Table 1), the addition of 5–40 µM Pb^2+^ significantly accelerated filament formation of wild-type Tau_244–372_ on the investigated time scale, compared with no Pb^2+^. Our control experiments verified that Pb^2+^ did not induce Tau filament formation in the absence of heparin on the investigated time scale of 8 h (Fig. S1).

**Table 1:** Kinetic parameters for fibril formation of human Tau fragment in the absence and in the presence of Pb^2+^ as determined by ThT binding assays at 37°C.

Molar ratio ( Pb^2+^/Tau)	*t*_50_ (min)	*F*(∞)
0	255±3	331±5
1∶2	143±21	321±4
1∶1	103±2	323±3
2∶1	142±3	280±5
3∶1	164±3	423±7
4∶1	146±3	276±4

Best-fit values of these kinetic parameters were derived from non-linear least squares modeling of the empirical Hill equation to the data plotted in Fig. 1A. Errors shown are standard errors of the mean.

Effect of Pb^2+^ on filament formation of wild-type Tau_244–372_ was further monitored *via* measurement of the time-dependent ANS fluorescence (Fig. 1B). Changes in ANS fluorescence are frequently used to detect the solvent-exposed hydrophobic clusters [17], [22]. As revealed by ANS fluorescence (Fig. 1B), the addition of 10 µM Pb^2+^ significantly accelerated the exposure of hydrophobic region and filament formation of wild-type Tau_244–372_ on the investigated time scale, compared with no Pb^2+^.

### Effect of Pb^2+^ on the secondary structures of Tau_244–372_

**Figure 2:**
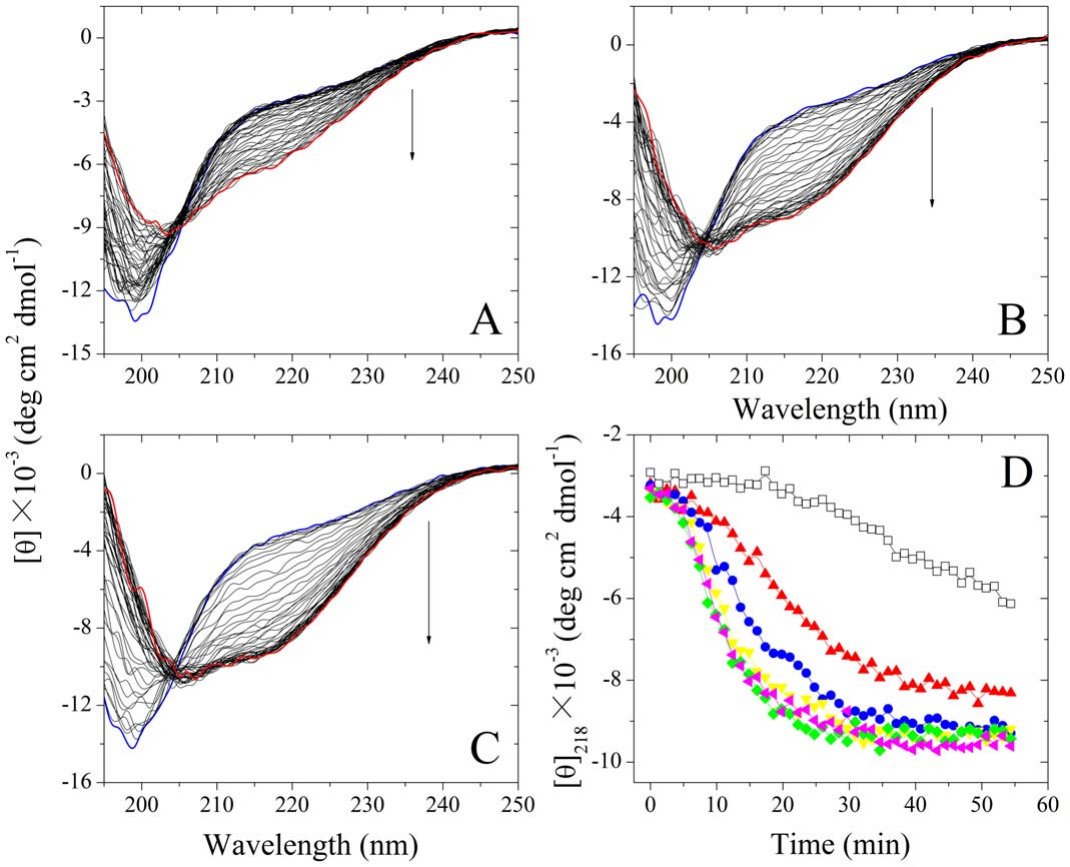
Far-UV CD spectra of Tau_244–372_ during fibril formation in the presence and absence of Pb^2+^ at 37°C. 10 µM Tau_244–372_ was incubated with 0–10 µM Pb^2+^ (A: 0 µM, B: 5 µM, and C: 10 µM). The arrows represented the incubation time increased gradually from 0 (the top, blue) to 54.4 min (the bottom, red). (D) Effect of Pb^2+^ on the relative change in the β-sheet content of Tau_244–372_ during fibril formation, studied by monitoring the CD signal at 218 nm. 10 µM Tau_244–372_ was incubated with 0–40 µM Pb^2+^ (black: 0 µM, red: 5 µM, blue: 10 µM, yellow: 20 µM, green: 30 µM, and magenta: 40 µM) in 30 mM phosphate buffer (pH 7.4) containing 1 mM DTT and 2.5 µM heparin.

CD spectroscopy was used to detect the conformational conversion of human Tau fragment during fibril formation in the presence and absence of Pb^2+^. Fig. 2A–C shows the far-UV CD spectra of wild-type Tau_244–372_ incubated with 0–10 µM Pb^2+^ at different incubation time points. As shown in Fig. 2A, at the beginning, the CD spectra measured for Tau_244–372_ in the absence of Pb^2+^ had a strong negative peak at 200 nm, indicative of a largely random coil structure. With the increase of the incubation time, the peak at 200 nm became smaller but the CD signal at 218 nm became larger gradually, indicative of β-sheet structure formed. As shown in Fig. 2B and 2C, such two signals of CD spectra of Tau_244–372_ in the presence of Pb^2+^ (5 and 10 µM) changed larger and faster than those in the absence of Pb^2+^ (Fig. 2A). Fig. 2D shows the effect of Pb^2+^ on the relative change in the β-sheet content of Tau_244–372_ during fibril formation, studied by monitoring the CD signal at 218 nm ([θ]_218_). As shown in Fig. 2D, in phosphate buffer, the value of [θ]_218_ of Tau_244–372_ aggregation in the absence of Pb^2+^ increased gradually from −2900 to −6100 deg⋅cm^2^⋅dmol^−1^ when the incubation time increased gradually from 0 to 54.4 min. In the presence of 10 µM Pb^2+^, however, the value of [θ]_218_ of Tau_244–372_ aggregation increased from −3200 to about −9000 deg⋅cm^2^⋅dmol^−1^ in the same incubation time range, reaching the maximum at 37.1 min. Similar phenomena were observed in the presence of 20, 30, and 40 µM Pb^2+^ in phosphate buffer (Fig. 2D). The maximal concentration of Pb^2+^ in phosphate buffer should be about 11 µM at 20°C and about 20 µM at 37°C according to the solubility product of PbHPO_4_. Consequently, it is not clear whether the traces in Fig. 2D are affected by insufficient lead solubility. We then performed CD measurements in sodium acetate buffer, in which Pb(CH_3_COO)_2_ is soluble. As shown in Fig. S2, in sodium acetate buffer, the value of [θ]_218_ of Tau_244–372_ aggregation in the absence of Pb^2+^ increased gradually from −4500 to −6300 deg⋅cm^2^⋅dmol^−1^ when the incubation time increased gradually from 0 to 36 min. In the presence of 10–40 µM Pb^2+^, however, the value of [θ]_218_ of Tau_244–372_ aggregation increased from −4300 to about −8000 deg⋅cm^2^⋅dmol^−1^ in the same incubation time range, reaching the maximum at 16 min. Therefore, as evidenced by CD spectroscopy (Figs. 2D and S2), fibrils formed by wild-type Tau_244–372_ in the presence of Pb^2+^ contain more β-sheet structure than the same amount of fibrils formed by the protein in the absence of Pb^2+^, and the addition of 5–40 µM Pb^2+^ significantly accelerated fibril formation of wild-type Tau_244–372_ on the investigated time scale. Clearly, the traces with molar ratios of Pb^2+^/Tau from 1∶1 to 4∶1 in the same buffer were similar (Figs. 2D and S2) not because of the same concentration of Pb^2+^ in solution, but due to the fact that one Pb^2+^ bound to one Tau monomer with sub-micromolar affinity (see below).

**Figure 3:**
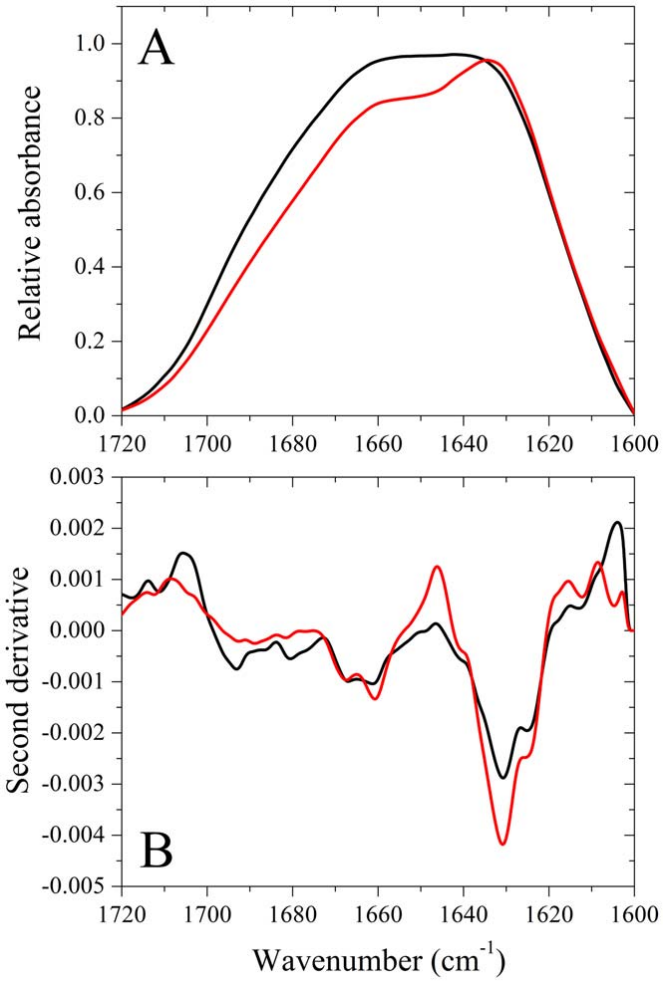
FTIR spectra of Tau_244–372_ fibrils formed in the presence and absence of Pb^2+^ at 37°C. (A) The FTIR spectra in the amide I′ region of Tau_244–372_ fibrils. (B) The second derivatives of the amide I′ bands of Tau_244–372_ fibrils. 200 µM Tau_244–372_ was incubated with 0–20 µM Pb^2+^ (black: 0 µM, and red: 20 µM) in 10 mM HEPES buffer (pH 7.4) containing 100 mM NaCl, 1 mM DTT, and 5 µM heparin, to produce fibrils.

FTIR was used to confirm the change in β-sheet structure of human Tau_244–372_ fibrils in the presence and absence of Pb^2+^. Fig. 3A shows the FTIR spectra in the amide I′ region of Tau_244–372_ fibrils and Fig. 3B displays the second derivatives. The amide I′ band at 1630 cm^−1^ is characteristic for β-sheet formed by amyloid fibrils [23]. As shown in Fig. 3B, compared with that in the absence of Pb^2+^, an increase of the band at 1630 cm^−1^ was clearly observed for Tau_244–372_ fibrils in the presence of 20 µM Pb^2+^, further supporting the conclusion reached by CD spectroscopy that fibrils formed by wild-type Tau_244–372_ in the presence of Pb^2+^ contain more β-sheet structure than the same amount of fibrils formed by the protein in the absence of Pb^2+^.

### Characterization of morphology of human Tau samples

**Figure 4:**
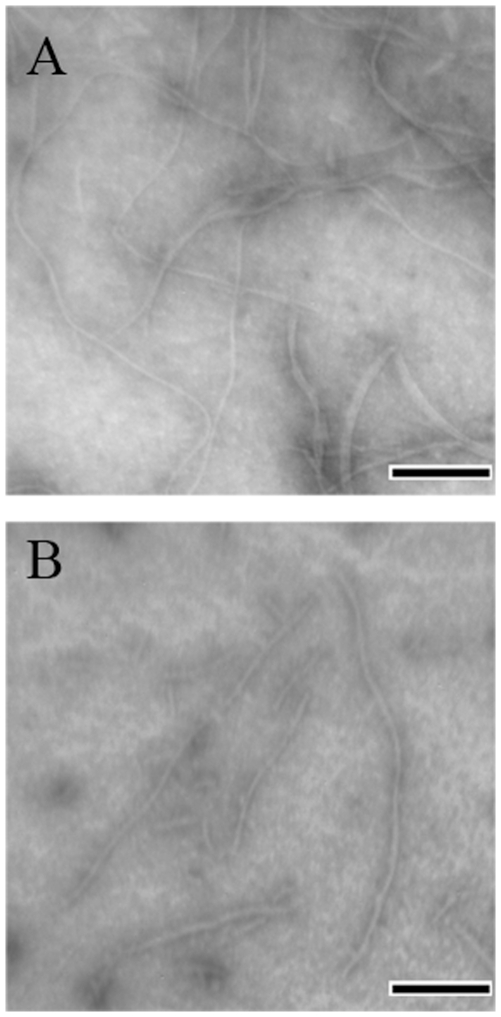
Transmission electron micrographs of Tau_244–372_ samples after incubation in the presence and absence of Pb^2+^. 10 µM Tau_244–372_ was incubated with 0–20 µM Pb^2+^ (A: 0 µM, and B: 20 µM) at 37°C for 8 h, in 10 mM HEPES buffer (pH 7.4) containing 100 mM NaCl, 1 mM DTT, and 2.5 µM heparin. A 2% (w/v) uranyl acetate solution was used to negatively stain the fibrils. The scale bars represent 200 nm.

TEM was used to study the morphology of human Tau samples incubated with 0–20 µM Pb^2+^. Our TEM studies confirmed the formation of fibrils by wild-type Tau_244–372_. As shown in Fig. 4A and 4B, long fibrils as well as short filaments were observed in both samples, indicating that the addition of Pb^2+^ had no significant effect on the morphology of Tau samples.

### His-330 and His-362 are key residues in the interaction of Pb^2+^ with Tau protein

**Figure 5:**
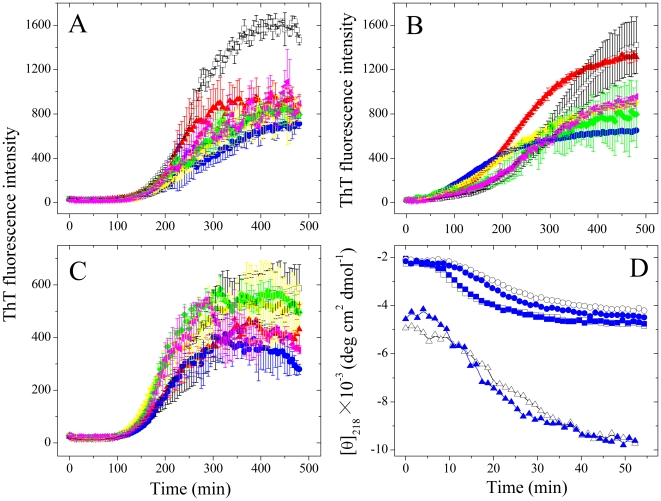
His-330 and His-362 are key residues in the interaction of Pb^2+^ with Tau_244–372_. 10 µM single mutants H330A (A) and H362A (B) or double mutant H330A/H362A (C) of Tau_244–372_ were incubated with 0–40 µM Pb^2+^ (black: 0 µM, red: 5 µM, blue: 10 µM, yellow: 20 µM, green: 30 µM, and magenta: 40 µM) in 10 mM HEPES buffer (pH 7.4) containing 100 mM NaCl, 1 mM DTT, 2.5 µM heparin, and 20 µM ThT, and ThT binding assays were carried out at 37°C. Data are expressed as mean ± S.D. (*n* = 3–4). (D) Effect of Pb^2+^ on the relative change in the β-sheet content of single mutants H330A (square) and H362A (circle) or double mutant H330A/H362A (triangle) during fibril formation, studied by monitoring the CD signal at 218 nm. 10 µM Tau_244–372_ was incubated with 0–10 µM Pb^2+^ (black: 0 µM, and blue: 10 µM) in 30 mM phosphate buffer (pH 7.4) containing 1 mM DTT and 2.5 µM heparin.

To determine the reason for the enhancing effect of Pb^2+^ on Tau fibrillization, histidine mutants of Tau_244–372_ were employed. There are five histidine residues in Tau_244–372_: His-268, His-299, His-329, His-330, and His-362. In this study, Tau_244–372_ mutants containing single, double, triple, and quintuple histidine mutations were designed, and ThT binding assays and far-UV CD experiments using such mutants were performed in order to provide information about the binding sites of Pb^2+^ in Tau protein and the role of histidine residues in Tau assembly. Fig. 5 shows the effects of Pb^2+^ on single mutants H330A and H362A and double mutant H330A/H362A of Tau_244–372_. Unlike wild-type Tau_244–372_, the presence of 5–40 µM Pb^2+^ had no obvious effects on fibrillization kinetics of single mutants H330A (Fig. 5A) and H362A (Fig. 5B) and double mutant H330A/H362A (Fig. 5C) except that 10 µM Pb^2+^ accelerated the aggregation of H362A to some extent (blue trace, Fig. 5B), and fibrils formed by such mutants in the absence and in the presence of Pb^2+^ contain similar amounts of β-sheet structure (Fig. 5D). However, the addition of 5–40 µM Pb^2+^ significantly accelerated filament formation of triple mutant H268A/H299A/H329A of Tau_244–372_ on the investigated time scale (data not shown). The above results suggest that His-330 and His-362 are key residues in the interaction of Pb^2+^ with Tau protein. It should be pointed out that the significant intensity variations between different measurements in Fig. 5 could reflect different fibril concentrations.

### Thermodynamics of the binding of Pb^2+^ to Tau protein

**Figure 6:**
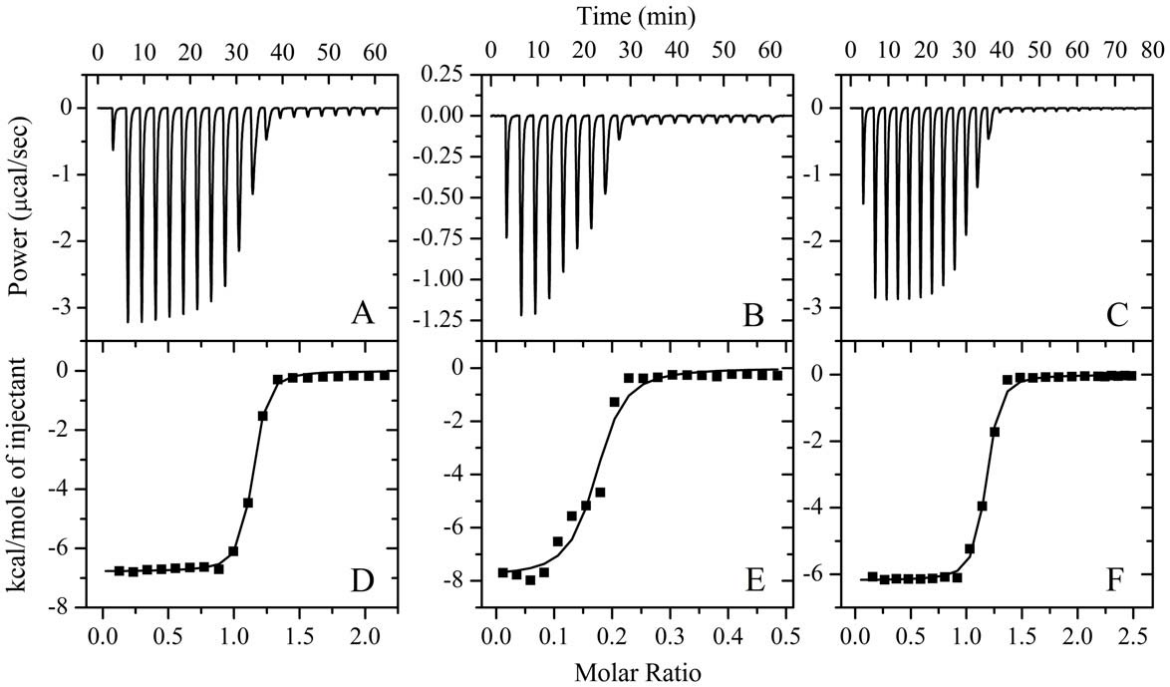
ITC profiles for the binding of Pb^2+^ to wild-type Tau_244–372_ and its mutants at 25.0°C. The top panels represent the raw data for sequential 10-µl injections of 1.5 mM Pb^2+^ into 150 µM wild-type Tau_244–372_ (A), 150 µM single mutant H362A (B), and 150 µM triple mutant H268A/H299A/H329A (C) in 50 mM Bis-Tris buffer (pH 7.4), respectively. The bottom panels (D, E, and F) show the plots of the heat evolved (kcal) per mole of Pb^2+^ added, corrected for the heat of Pb^2+^ dilution, against the molar ratio of Pb^2+^ to Tau_244–372_. The data (solid squares) were best fitted to a single set of identical sites model and the solid lines represented the best fit.

ITC provides a direct route to the complete thermodynamic characterization of non-covalent, equilibrium interactions [16], [18], [22], and DTT concentrations as low as 1 mM can cause severe baseline artifacts due to background oxidation during the titration. Therefore ITC was used to measure the binding affinity of Pb^2+^ to Tau protein in the absence of DTT. ITC profiles for the binding of Pb^2+^ to wild-type Tau_244–372_ and its histidine mutants at 25.0°C are shown in Figs. 6 and S3. The top panels show representatively raw ITC curves resulting from the injections of Pb^2+^ into a solution of wild-type Tau_244–372_ (Fig. 6A), single mutants H362A (Fig. 6B) and H330A (Fig. S3A), double mutant H330A/H362A (Fig. S3B), and triple mutant H268A/H299A/H329A (Fig. 6C). The titration curves show that Pb^2+^ binding to wild-type Tau_244–372_ and its histidine mutants were exothermic, resulting in negative peaks in the plots of power *versus* time. The bottom panels show the plots of the heat evolved per mole of Pb^2+^ added, corrected for the heat of Pb^2+^ dilution, against the molar ratio of Pb^2+^ to wild-type Tau_244–372_ (Fig. 6D), H362A (Fig. 6E), H330A (Fig. S3C), H330A/H362A (Fig. S3D), and H268A/H299A/H329A (Fig. 6F). The calorimetric data were best fit to a model assuming a single set of identical sites. The thermodynamic parameters for the binding of Pb^2+^ to Tau_244–372_ are summarized in Table 2. As shown in Table 2, one Pb^2+^ bound to one wild-type Tau_244–372_ (or one triple mutant H268A/H299A/H329A) molecule with a dissociation constant of 0.217 µM (or 0.286 µM). The binding affinity of Pb^2+^ to single histidine mutant H362A was significantly lower than that of wild-type Tau_244–372_, with a dissociation constant of 0.546 µM, and a weak binding reaction for Pb^2+^ with single histidine mutant H330A was observed. No binding reaction for Pb^2+^ with double histidine mutant H330A/H362A or histidine-less mutant was detected by ITC (Table 2), demonstrating that His-330 and His-362 are key residues in the interaction of Pb^2+^ with Tau protein. Our ITC data (Table 2) clearly indicated that at physiological pH, one Pb^2+^ bound to one Tau monomer *via* interaction with His-330 and His-362, with sub-micromolar affinity.

**Table 2:** Thermodynamic parameters for the binding of Pb^2+^ to Tau_244–372_ (or full-length Tau protein) as determined by ITC at 25.0°C.

Tau_244–372_	*K*_d_ (µM)	*n*	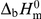 (kcal mol^−1^)	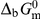 (kcal mol^−1^)	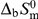 (cal mol^−1^ K^−1^)
WT	0.217±0.029	1.090±0.003	−6.78±0.04	−9.08±0.08	7.73±0.39
H330A	82±48	0.59±0.32	−0.31±0.21	−5.79±0.36	17.7±1.8
H362A	0.546±0.020	0.165±0.047	−7.85±0.29	−8.53±0.22	2.32±1.72
DM	NB	–	–	–	–
TM	0.286±0.042	1.130±0.005	−6.19±0.05	−8.92±0.08	9.17±0.45
Histidine-less	NB	–	–	–	–
Full-length Tau	0.29±0.16	2.72±0.06	−7.86±0.30	−8.91±0.32	3.55±2.05

Thermodynamic parameters, Kd, 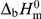, and n, were determined using a single set of identical sites model. The standard molar binding free energy (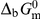) and the standard molar binding entropy (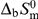) for the binding reaction were calculated using Equations 2 and 3 respectively.

The buffer used was 50 mM Bis-Tris buffer (pH 7.4). Errors shown are standard errors of the mean.

WT, wild-type Tau_244–372_; DM, double mutant H330A/H362A of Tau_244–372_; TM, triple mutant H268A/H299A/H329A of Tau_244–372_; Histidine-less, histidine-less mutant H268A/H299A/H329A/H330A/H362A.

NB, no binding observed in the present conditions.

## Discussion

Because heavy metals persist in the environment (they cannot be destroyed biologically) and are carcinogenic to human being, pollution by heavy metals poses a great potential threat to the environment and human health [24], [25]. Among them, lead is a potent neurotoxin for human being especially for the developing children due to a causal link between low-level chronic exposure to lead and deficiencies in intelligence quotients in children [26]–[28]. The source of lead used in our daily life contains mining and smelting of metalliferous ores, burning of leaded gasoline, municipal sewage, industrial wastes, paints, and some food [25], [29], [30]. Some early studies have indicated that exposure to lead in early life could have long-term effects and thereby significantly increases the risk of developing Alzheimer disease in later years [31]–[33]. Recent studies in rodents have shown that exposure to lead during brain development is able to predetermine the expression and regulation of amyloid precursor protein and its amyloid β product in old age [8], [34]. It has been reported that exposure to lead disturbs the balance between amyloid β production and elimination [35]. Furthermore, the expression of Alzheimer disease-related genes and their transcriptional regulator are elevated in 23-year-old monkeys exposed to lead as infants leading to an Alzheimer disease-like pathology in the aged monkeys [10]. Chronic lead exposure also affects granule cell morphology in lead-exposed rats, whose dendrites frequently appear dystrophic, similar to those present in Alzheimer disease [36]. Because Pb^2+^ at high concentrations has been found in the brains of patients with Alzheimer disease [2] and with diffuse neurofibrillary tangles with calcification [11], we wanted to know whether Pb^2+^ plays a role in the pathology of Alzheimer disease through enhancing Tau filament formation.

In this paper the concentration of Pb^2+^ used was 5–40 µM because of the following reasons. Firstly, it has been demonstrated that there is no significant cytotoxicity to SH-SY5Y cells for 1–50 µM of Pb^2+^ at either 48 or 72 h [35]. Secondly, the concentration of Pb^2+^ we used is one order of magnitude higher than that considered as lead poisoning by public health authorities in the United States and France [37]. In addition, the concentration of Tau protein we used is of the same order of magnitude as that of endogenous Tau present in human brain [38]. We demonstrated for the first time that the fibrillization of human Tau protein was accelerated by exposure to 5–40 µM Pb^2+^ *via* interaction with His-330 and His-362, with sub-micromolar affinity. In other words, His-330 and His-362 are key residues in the interaction of Pb^2+^ with Tau protein. Moreover, fibrils formed by human Tau protein in the presence of 5–40 µM Pb^2+^ contained more β-sheet structure than the same amount of fibrils formed by the protein in the absence of Pb^2+^. In other words, exposure to 5–40 µM Pb^2+^ enhanced the conversion of random coil structure into β-sheet structure and thereby accelerated the fibrillization of human Tau protein. Our results suggest the possible involvement of Pb^2+^ in the pathogenesis of Alzheimer disease and provide critical insights into the mechanism of lead toxicity.

For ITC experiments Tau_244–372_ was dialyzed overnight at 4°C in the absence of DTT. It has been reported that similar conditions (Tau_244–394_ is dialyzed for 7 days at 20°C in the absence of DTT) result in the oxidation of the two cysteines and lead to the formation of compact monomers and a minor population of dimers [39]. Consequently, it is not clear which Tau species is analyzed in the ITC measurements. We then turned to native gel electrophoresis. As shown in Fig. S4, we observed only one population of monomers, the extended Tau_244–372_ monomers, but neither dimers nor compact monomers, in the ITC experimental conditions. Lane 2 serves as a standard where Tau_244–372_ was in the presence of DTT, resulting in a purely monomeric population (Fig. S4). Clearly, the extended Tau_244–372_ monomers were analyzed in our ITC measurements.

The above experiments were conducted using Tau_244–372_ with four repeats, but filaments in Alzheimer disease contain full-length Tau protein. There are ten histidine residues in full-length human Tau protein and five histidine residues in Tau_244–372_. Our additional ITC experiments (Fig. S5) indicated that in the absence of DTT, Pb^2+^ bound to full-length human Tau protein with a sub-micromolar affinity (0.29±0.16 µM) similar to Tau_244–372_, but with a binding stoichiometry (2.72±0.06) remarkably larger than Tau_244–372_ (Table 2). Therefore, it is possible that other histidine residues (or other residues) beyond the region can bind to Pb^2+^ as well.

In conclusion we have shown that: (i) the addition of micromolar concentrations of Pb^2+^ significantly accelerates the exposure of hydrophobic region and filament formation of human Tau protein; (ii) fibrils formed by human Tau protein in the presence of micromolar concentrations of Pb^2+^ contain more β-sheet structure than the same amount of fibrils formed by the protein in the absence of Pb^2+^; (iii) the fibrillization of human Tau protein is promoted by exposure to Pb^2+^ *via* interaction with His-330 and His-362, with sub-micromolar affinity. Information obtained here can enhance our understanding of how low levels of inorganic lead interact with microtubule-associated protein Tau in pathological environments and thereby play a role in the pathology of Alzheimer disease.

## Supporting Information

Figure S1 **Pb^2+^ alone did not induce Tau filament formation.** 10 µM Tau_244–372_ was incubated with 0–10 µM Pb^2+^ (black: 0 µM, and blue: 10 µM) in 10 mM HEPES buffer (pH 7.4) containing 100 mM NaCl, 1 mM DTT, 2.5 µM heparin, and 20 µM ThT, or incubated with 10 µM Pb^2+^ (red) in 10 mM HEPES buffer (pH 7.4) containing 100 mM NaCl, 1 mM DTT, and 20 µM ThT. ThT binding assays were carried out at 37°C, and all experiments were repeated at least twice.
(DOC)

Figure S2 **Effect of Pb^2+^ on the relative change in the β-sheet content of Tau_244–372_ during fibril formation at 37°C, studied by monitoring the CD signal at 218 nm.** 10 µM Tau_244–372_ was incubated with 0–40 µM Pb^2+^ (black: 0 µM, red: 5 µM, blue: 10 µM, yellow: 20 µM, green: 30 µM, and magenta: 40 µM) in 50 mM sodium acetate buffer (pH 7.4) containing 1 mM DTT and 2.5 µM heparin.
(DOC)

Figure S3 **ITC profiles for the binding of Pb^2+^ to Tau_244–372_mutants at 25.0°C.** The top panels represent the raw data for sequential 10-µl injections of 1.5 mM Pb^2+^ into 150 µM single mutant H330A (A) and 150 µM double mutant H330A/H362A (B) in 50 mM Bis-Tris buffer (pH 7.4), respectively. The bottom panels (C and D) show the plots of the heat evolved (kcal) per mole of Pb^2+^ added, corrected for the heat of Pb^2+^ dilution, against the molar ratio of Pb^2+^ to Tau_244–372_. The data (solid squares) were best fitted to a single set of identical sites model and the solid lines represented the best fit.
(DOC)

Figure S4 **Native gel electrophoresis of Tau_244–372_.** Lane 1, Tau_244–372_ in the absence of DTT (oxidative conditions). Lane 2, Tau_244–372_ in the presence of 1 mM DTT (reducing conditions). Only one population of monomers, the extended Tau_244–372_ monomer (M), was visible in lanes 1 and 2. Freshly purified wild-type Tau_244–372_ was dialyzed against 50 mM Bis-Tris buffer (pH 7.4) containing 1 mM EDTA and 100 mM NaCl, overnight at 4°C and then dialyzed against 50 mM Bis-Tris buffer (pH 7.4) containing 100 mM NaCl extensively to remove EDTA. The samples were mixed with 2× loading buffer and separated by 15% native PAGE. Gel was stained by Coomassie Blue G250.
(DOC)

Figure S5 **ITC profiles for the binding of Pb^2+^ to full-length Tau protein at 25.0°C.** The panel A represents typical calorimetric titration of full-length Tau (150 µM) with Pb^2+^ (1.0 mM) in 50 mM Bis-Tris buffer (pH 7.4). The first injection (5 µl) was followed by 19 injections of 10 µl. The panel *B* shows the plots of the heat evolved (kcal) per mole of Pb^2+^ added, corrected for the heat of Pb^2+^ dilution, against the molar ratio of Pb^2+^ to full-length Tau. The data (solid squares) were best fitted to a single set of identical sites model and the solid lines represented the best fit.
(DOC)
