# Supplementary figures and images for: Fibrillization of Human Tau Is Accelerated by Exposure to Lead via Interaction with His-330 and His-362
Source: PLoS One. 2011 Sep 26;6(9):e25020. doi: 10.1371/journal.pone.0025020 (PMC3180286; doi:10.1371/journal.pone.0025020)

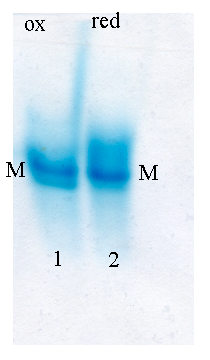

Supplement: Figure S4 — Native gel electrophoresis of Tau244–372. Lane 1, Tau244–372 in the absence of DTT (oxidative conditions). Lane 2, Tau244–372 in the presence of 1 mM DTT (reducing conditions). Only one population of monomers, the extended Tau244–372 monomer (M), was visible in lanes 1 and 2. Freshly purified wild-type Tau244–372 was dialyzed against 50 mM Bis-Tris buffer (pH 7.4) containing 1 mM EDTA and 100 mM NaCl, overnight at 4°C and then dialyzed against 50 mM Bis-Tris buffer (pH 7.4) containing 100 mM NaCl extensively to remove EDTA. The samples were mixed with 2× loading buffer and separated by 15% native PAGE. Gel was stained by Coomassie Blue G250. (DOC) [file pone.0025020.s004.doc]
