# Supplementary material for: Fibrillization of Human Tau Is Accelerated by Exposure to Lead via Interaction with His-330 and His-362
Source: PLoS One. 2011 Sep 26;6(9):e25020. doi: 10.1371/journal.pone.0025020 (PMC3180286; doi:10.1371/journal.pone.0025020)
Supplement: Supplementary file 2 [file pone.0025020.s001.doc]

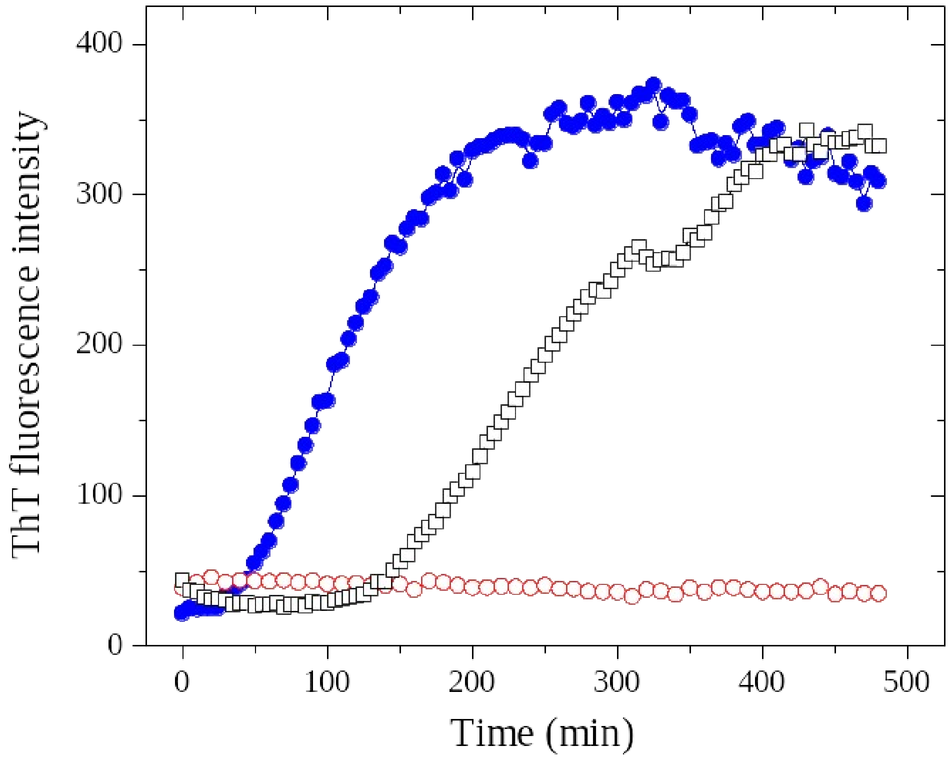
ThT fluorescence intensity
400
300
200
100
0
0
100
200
300
400
500
Time (min)
