# Supplementary material for: Fibrillization of Human Tau Is Accelerated by Exposure to Lead via Interaction with His-330 and His-362
Source: PLoS One. 2011 Sep 26;6(9):e25020. doi: 10.1371/journal.pone.0025020 (PMC3180286; doi:10.1371/journal.pone.0025020)
Supplement: Supplementary file 3 [file pone.0025020.s002.doc]

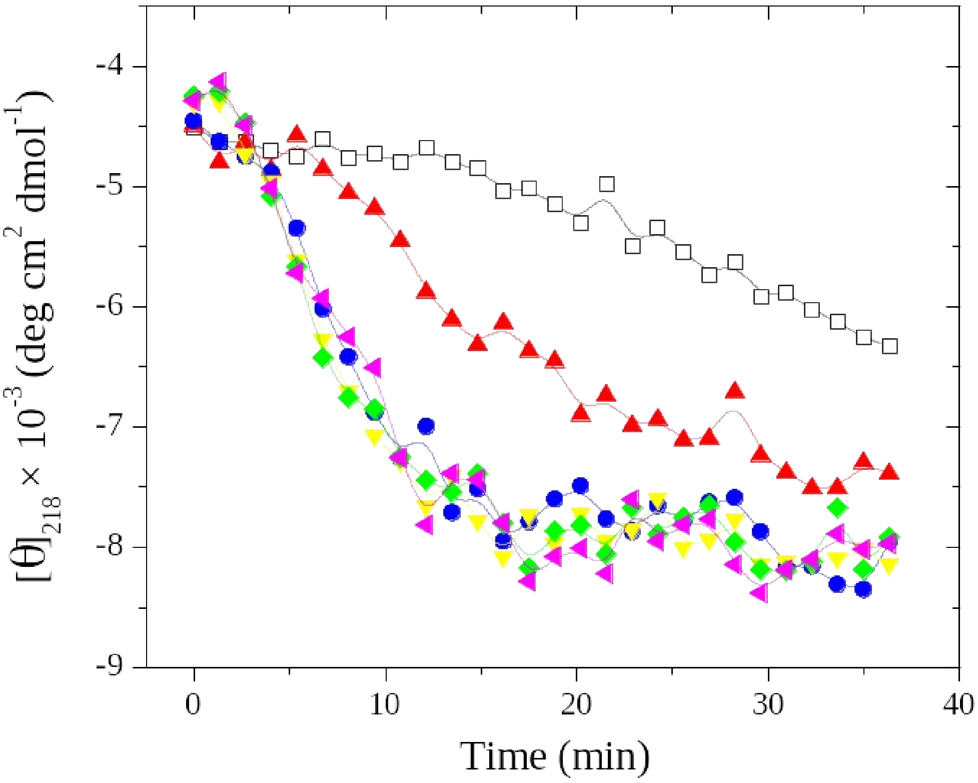

$[\Theta]_{218} \times 10^{-3}$ (deg cm^2 dmol^{-1})
-4
-5
-6
-7
-8
-9
0
10
20
30
40
Time (min)
