# Supplementary material for: Fibrillization of Human Tau Is Accelerated by Exposure to Lead via Interaction with His-330 and His-362
Source: PLoS One. 2011 Sep 26;6(9):e25020. doi: 10.1371/journal.pone.0025020 (PMC3180286; doi:10.1371/journal.pone.0025020)
Supplement: Supplementary file 4 [file pone.0025020.s003.doc]

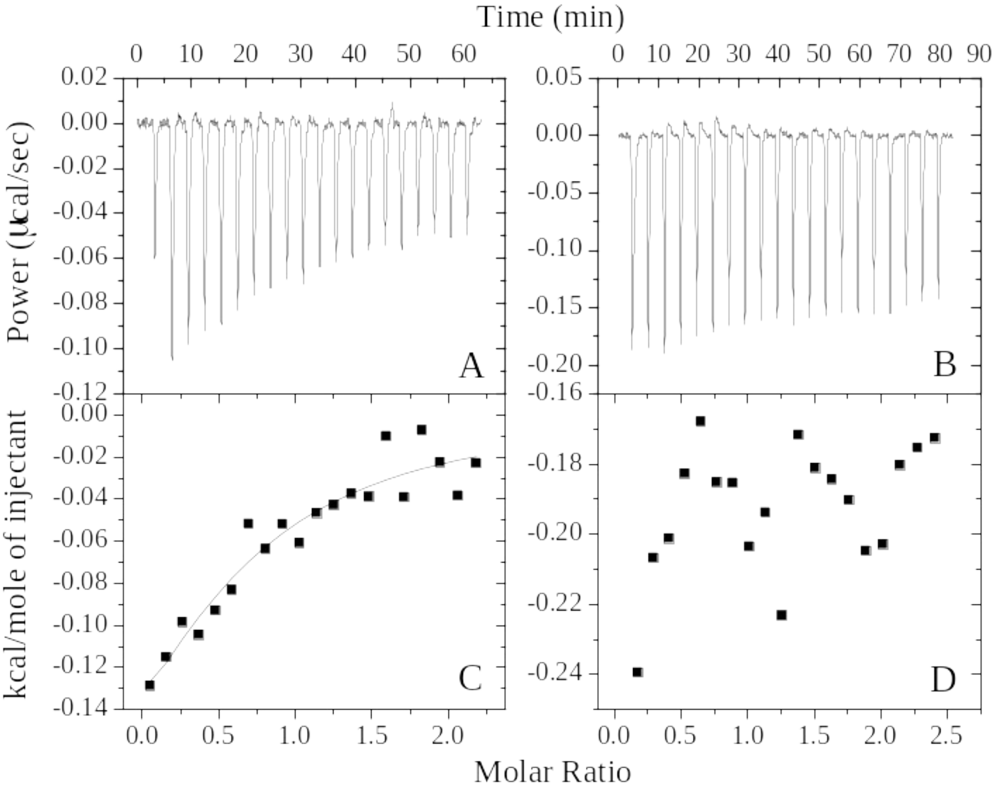
Time (min)
Power (μcal/sec)
kcal/mole of injectant
Molar Ratio
A
B
C
D
