# Supplementary material for: Fibrillization of Human Tau Is Accelerated by Exposure to Lead via Interaction with His-330 and His-362
Source: PLoS One. 2011 Sep 26;6(9):e25020. doi: 10.1371/journal.pone.0025020 (PMC3180286; doi:10.1371/journal.pone.0025020)
Supplement: Supplementary file 5 [file pone.0025020.s005.doc]

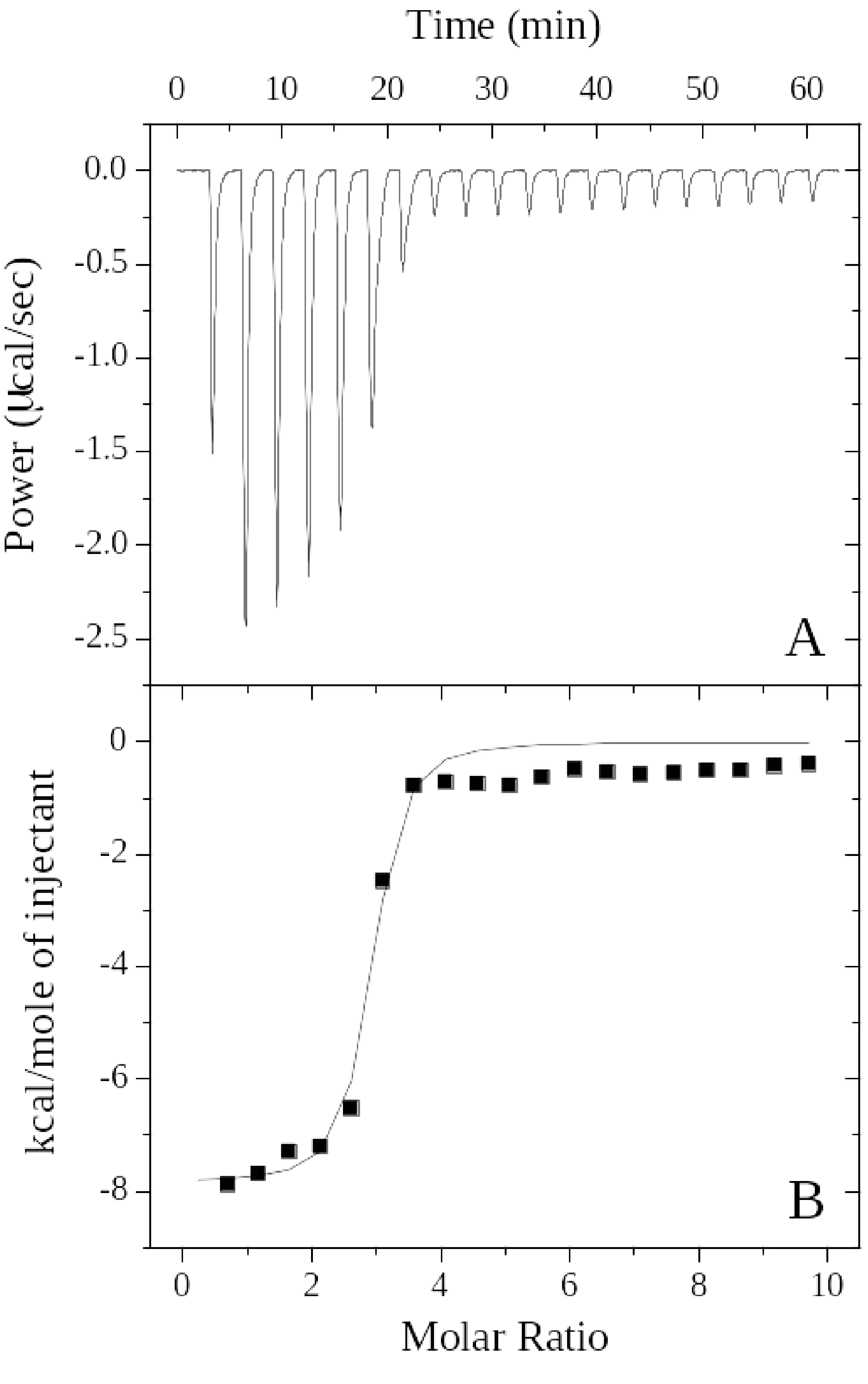
Time (min)
0
10
20
30
40
50
60
Power (μcal/sec)
0.0
-0.5
-1.0
-1.5
-2.0
-2.5
A
kcal/mole of injectant
0
-2
-4
-6
-8
B
0
2
4
6
8
10
Molar Ratio
